# Synergistic effects of aerobic exercise and cognitive training on cognition, physiological markers, daily function, and quality of life in stroke survivors with cognitive decline: study protocol for a randomized controlled trial

**DOI:** 10.1186/s13063-017-2153-7

**Published:** 2017-08-31

**Authors:** Ting-ting Yeh, Ching-yi Wu, Yu-wei Hsieh, Ku-chou Chang, Lin-chien Lee, Jen-wen Hung, Keh-chung Lin, Ching-hung Teng, Yi-han Liao

**Affiliations:** 1 School of Occupational Therapy, College of Medicine, National Taiwan University, Taipei, Taiwan; 2 Department of Occupational Therapy and Graduate Institute of Behavioral Sciences, College of Medicine, Chang Gung University, Taoyuan, Taiwan; 3 Healthy Aging Research Center, Chang Gung University, Taoyuan, Taiwan; 4 Department of Physical Medicine and Rehabilitation, Chang Gung Memorial Hospital at Linkou, Taoyuan, Taiwan; 5 Department of Neurology, Chang Gung Memorial Hospital, Kaohsiung, Taiwan; 6 Department of Physical Medicine and Rehabilitation, Cheng Hsin General Hospital, Taipei, Taiwan; 7 Department of Rehabilitation, Chang Gung Memorial Hospital-Kaohsiung Medical Center, Chang Gung University, College of Medicine, Kaohsiung, Taiwan; 8 Division of Occupational Therapy, Department of Physical Medicine and Rehabilitation, National Taiwan University Hospital, Taipei, Taiwan

**Keywords:** Stroke, Cognitive rehabilitation, Aerobic exercise, Cognitive training, Sequential training

## Abstract

**Background:**

Aerobic exercise and cognitive training have been effective in improving cognitive functions; however, whether the combination of these two can further enhance cognition and clinical outcomes in stroke survivors with cognitive decline remains unknown. This study aimed to determine the treatment effects of a sequential combination of aerobic exercise and cognitive training on cognitive function and clinical outcomes.

**Methods/design:**

Stroke survivors (*n* = 75) with cognitive decline will be recruited and randomly assigned to cognitive training, aerobic exercise, and sequential combination of aerobic exercise and cognitive training groups. All participants will receive training for 60 minutes per day, 3 days per week for 12 weeks. The aerobic exercise group will receive stationary bicycle training, the cognitive training group will receive cognitive-based training, and the sequential group will first receive 30 minutes of aerobic exercise, followed by 30 minutes of cognitive training. The outcome measures involve cognitive functions, physiological biomarkers, daily function and quality of life, physical functions, and social participation. Participants will be assessed before and immediately after the interventions, and 6 months after the interventions. Repeated measures of analysis of variance will be used to evaluate the changes in outcome measures at the three assessments.

**Discussion:**

This trial aims to explore the benefits of innovative intervention approaches to improve the cognitive function, physiological markers, daily function, and quality of life in stroke survivors with cognitive decline. The findings will provide evidence to advance post-stroke cognitive rehabilitation.

**Trial registration:**

ClinicalTrials.gov, NCT02550990. Registered on 6 September 2015.

**Electronic supplementary material:**

The online version of this article (doi:10.1186/s13063-017-2153-7) contains supplementary material, which is available to authorized users.

## Background

Post-stroke cognitive impairment is a major problem that affects up to 70% of stroke survivors [1–3]. Persistent cognitive impairment after stroke not only causes deterioration in patients’ ability to relearn motor skills due to memory problems or poor judgment, but also significantly impairs the activities of daily living (ADL) and quality of life (QOL) [4].

Post-stroke cognitive impairment and rehabilitation have recently been identified as the top research priority for post-stroke survivors [5]. Current evidence indicates that targeted cognitive rehabilitation after stroke, including exercise training [6–8] or cognitive training [9, 10], provides potential means of promoting cognitive function. A recent overview of the evidence for the effectiveness of cognitive rehabilitation for stroke survivors suggests that cognitive training targeting specific cognitive domains has some positive effects [11]. For example, Starovasnik Žagavec et al. [12] studied the effect of computer-based cognitive rehabilitation on work-active patients after stroke and reported a moderate to strong effect on the divided attention and a mild effect on the alertness. They suggested that the attention system can be rehabilitated by training the reaction speed and learning the awareness and selection of suitable stimuli. Another randomized controlled trial examined the effects of 5 weeks working memory training in stroke survivors and found training effects on attention and subjective experience of cognitive functioning in daily living (as measured by the Cognitive Failure Questionnaire) [13]. These results provide evidence that intensive cognitive training can improve cognitive function in stroke survivors. A recent study suggests that the increased resting-state functional connectivity of the hippocampus with the frontal and parietal lobes may be an important mechanism of cognitive recovery after stroke [14].

Another important approach that has been proposed to enhance cognitive function is aerobic exercise. In addition to the well-known benefits of exercise in improving physical function and reducing the risk of secondary complications, exercise has been found to lower the risk of developing Alzheimer’s disease and related cognitive disorders [15]. Cumming and colleagues conducted a systematic review and reported that increased physical activity after stroke improves cognitive functions [16]. It has been suggested that aerobic exercise may increase the arousal level and reduce depressive symptoms, which in turn leads to better cognitive function [16]. The improvement in cognitive function after exercise could be induced by the upregulation of neurotrophic and vascular growth factors, which may facilitate neurogenesis, angiogenesis, and synaptic plasticity of the hippocampus and other cognition-related cortical areas [17, 18]. It has been shown that the neurotrophin brain-derived neurotrophic factor (BDNF) plays a role in regulating synaptic connectivity [19]. Recently, several clinical studies have demonstrated that BDNF may be act as a biomarker of memory and general cognitive function in healthy adults [20], and in patients with schizophrenia [21] and Parkinson’s disease [22]. Although BDNF has been widely investigated, the mechanism of action and the biological correlates of cognitive rehabilitation in patients with stroke are still unknown. Also, it remains unclear whether BDNF serum levels may be used as a biomarker of cognitive training in patients with stroke.

Current evidence is insufficient to establish clinical practice recommendations due to the small sample sizes, unclear treatment details and different measurements of the outcome variable. There is urgent need to perform a high-quality randomized controlled trial (RCT) that is adequately powered, using the optimum intervention and validated outcome measurements. Furthermore, few of the prior studies included outcome measures of changes in physiological markers, ADLs, and QOL. Growing evidence suggests that a hybrid combination of aerobic exercise and cognitive training may provide additional benefits in cognitive performance that go beyond the effects of a single type of training in animals [23], community-dwelling elderly [24, 25], and stroke survivors [26]. A systematic review of combining exercise and cognitive intervention in older adults suggests that exercise sessions delivered *before* the cognitive training session prepares the brain for the compensatory recruitment process in the subsequent cognitive training sessions [25]. Aerobic exercise before cognitive training may increase arousal level, facilitate neurogenesis and enhance memory consolidation, which may benefit the memory retrieval and cognitive task performance that follows [27, 28]. Aerobic exercise training combined with cognitive training might be a plausible intervention to augment cognitive function and intensify rehabilitation outcomes.

We are conducting a single-blind, adequately powered, high-quality RCT to test the hypothesis that a sequential combination of aerobic exercise and cognitive training will promote cognitive performance in stroke survivors with cognitive impairment. Specifically, we aim to determine the treatment effects of the hybrid training, compared with exercise or cognitive training alone, on: (1) cognitive function and (2) physiological markers, daily function, quality of life, physical function, and social participation.

## Methods/design

### Study design and setting

**Fig. 1:**
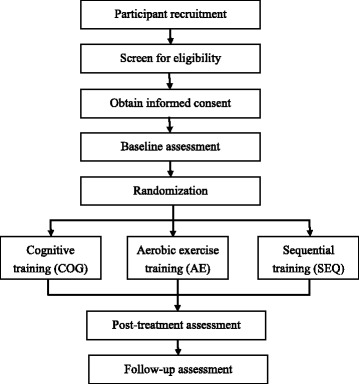
Flow diagram of the study

**Fig. 2:**
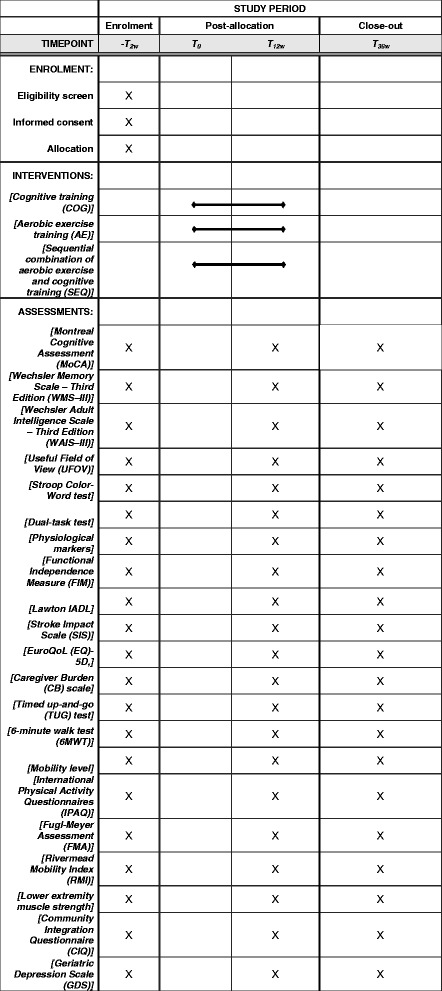
SPIRIT figure

This study will be a single-blind, multisite, randomized controlled trial. After signing the informed consent, participants will be stratified by Mini-Mental State Examination (MMSE) scores and randomly assigned to the cognitive training (COG), aerobic exercise (AE), or sequential combination of aerobic exercise and cognitive training (SEQ) groups (Fig. 1) by an independent research assistant. The randomization scheme will be generated with the web-based randomization tool (available at http://www.randomizer.org/), applying block randomization to achieve three groups with a ratio of 1:1:1. All participants will receive 36 training sessions over a 3-month period. The participants will be assessed before and immediately after the intervention programs, and 6 months after the intervention programs. The evaluator for all assessments (before, after, and follow up) will be blinded to the group allocation. Figure 2 illustrates the timing of all trial processes (Additional file 1)﻿.

### Sample size

No published research to date has investigated the effects of combining aerobic exercise with cognitive training on cognitive functions in patients with stroke; therefore, the sample size calculation was based on the effects of a similar study conducted by Kim et al. [29]. The study investigated the effects of 4 weeks of cognitive, combined, motor dual-task training on cognitive and motor function in stroke survivors. The Stroop test, Timed Up and Go (TUG) test, 10-Meter Walk Test (10MWT), Figure-of-8 Walk Test (F8WT) and gait speed were used to measure cognitive and motor abilities and were evaluated three times (before and after training and at the 2-week follow up). The results showed that compared to the single-task training, the active control group, dual-task training improves cognitive and motor functions in stroke survivors, and these training benefits were maintained for 2 weeks.

A priori power analysis (G*Power software 3.1) was used to estimate the number of participants needed for this project. On the basis of Kim’s work [29], the estimated sample size requirement is 20 patients for each group in a three-group study design given the lowest effect size (*f* = 0.30), power of 0.80, and two-sided type I error of 0.05. Allowing for 20% attrition at the 6-month follow up, we plan to recruit 25 participants for each group, resulting in a sample size of 75 participants.

### Participant recruitment

This proposed study will be conducted in the outpatient units of the Departments of Neurology and Rehabilitation in Chang Gung Memorial Hospitals at five branches (Taoyuan, Chiayi, Kaohsiung, Keelung and Taipei) in Taiwan. This trial was approved by the centralized ethics committee of Chang Gung Memorial Hospital Human Research Ethics Board (Institutional Review Board (IRB)# 104-4892A3). Participants will be recruited via the clinic database according to diagnosis and medical referral; interested participants will meet with the study personnel to receive all necessary information. The participants will resolve any uncertainties and be invited to sign informed consent after an assessment of eligibility criteria.

### Inclusion and exclusion criteria

Participants eligible for the study must comply with all of the following criteria before randomization: (1) unilateral ischemic or hemorrhagic stroke occurring at least 6 months before enrolment; (2) age between 20 and 90 years [30]; (3) MMSE score ≥ 19; (4) Montreal Cognitive Assessment (MoCA) < 26; (5) self-reported or informant-reported memory or cognitive complaints or Clinical Dementia Rating Scale (CDR) score ≤ 0.5; (6) able to follow the study instruction; (7) adequate cardiopulmonary function to perform aerobic exercise; and (8) able to walk with or without assistive devices. Patients who have one or more of the following criteria will be excluded from the study: (1) unstable medical history (e.g., recent myocardial infarction) that might limit participation; (2) other neurologic disorders (e.g., Parkinson’s disease, amyotrophic lateral sclerosis, multiple sclerosis); and (3) current participation in another interventional trial.

### Interventions

Participants will be randomized to the COG, AE, or SEQ groups. The experimental intervention will be added to the existing conventional neurorehabilitative treatment as prescribed by each clinic. Conventional treatment, including physiotherapy and occupational therapy, will be documented. The total conventional treatment time will be an hour for all participants across all investigational sites to ensure the amount of conventional neurorehabilitative treatment time is consistent. A licensed occupational therapist will train the interventionists to ensure standardized treatment delivery. After the initial training, the occupational therapist will test the interventionists to determine whether they are capable of conducting the standardized intervention protocol. Periodic on-site supervision will be administered to prevent protocol deviations. The interventionists will use standardized protocol calendars to record the treatment dosage and participants’ progress and responses during each intervention session. The standardized protocol calendars will be reviewed by the principle investigator to ensure adequate “dose” of treatment is received and consistent dose of treatment across sites.

### Cognitive training (COG)

Computer-assisted cognitive training (i.e., the BrainHQ program) will be used to facilitate several cognitive functions in the COG group. The specific domains of cognitive function to be targeted are attention, recognition, color and shape identification, calculation, visual perception, visuospatial processing, and executive function. Computer-assisted cognitive training allows automation of many cognitive training procedures, making it possible to improve stimulation quality, increase patient record reliability, and optimize performance. Participants will perform a variety of tasks designed to enhance different types of cognitive functions. The training program will be adjusted automatically and continuously according to each participant’s level of performance. Each cognitive intervention session will last 60 minutes.

### Aerobic exercise training (AE)

The AE group will perform progressive resistive stationary bicycle training as in Quaney et al. [8]. The participants will first perform 10 minutes of warm up, followed by 45 minutes of resistive aerobic exercise and 5 minutes of cool down. The target heart rate during the aerobic period will be 40–70% of the patient’s maximal heart rate, calculated as (208 − 0.7 × age) [31]. The exercise intensity will be progressed as the participants improve their performance throughout practice. Vital signs and the Borg Perceived Exertion Scale [32] will be monitored and recorded during each exercise session. The mean heart rate will be recorded. Each aerobic exercise session will last for 60 minutes.

### Sequential combination of aerobic exercise and cognitive training (SEQ)

Participants in the SEQ group will receive aerobic exercise training and computer-assisted cognitive training combined. Each session will contain 30 minutes of the aerobic exercise, followed by 30 minutes of cognitive training. The participants will first perform 3 minutes of warm up, followed by 25 minutes of aerobic resistive exercise, and end with 2 minutes of cool down for a total of 30 minutes of exercise training. The aerobic exercise intensity will be similar to the AE group. Vital signs, Borg Perceived Exertion Scale [32], and mean heart rate will be monitored and recorded. After the aerobic exercise, the participants will take part in 30 minutes of cognitive training similar to the training paradigm used in the COG group.

### Outcome assessment

Outcomes will be measured once before the intervention, at 12 weeks after the intervention, and at 6 months after the intervention, with each assessment lasting 1 to 1.5 hours. Assessments will be conducted by a trained and experienced therapist who is blinded to group allocation. None of the assessors will participate in the training intervention.

Some standardized measures will be used to assess changes. At baseline, additional assessments of disease severity of the participants will be administered for classification of the included patients, including the National Institutes of Health Stroke Scale (NIHSS), Fugl-Meyer Assessment (FMA), Modified Rankin Scale (MRS), and MMSE.

### Primary outcome measure: cognitive functions

The Montreal Cognitive Assessment (MoCA) is a 30-point test that examines general cognitive functions, including memory, attention, language, visuospatial ability and execution, naming, delayed recall, abstraction, and orientation. The MoCA is a feasible tool to evaluate the global cognitive function in a large population of patients with stroke [33]. Good-to-excellent reliability and validity have been established in patients with cerebrovascular disease [34].

The Wechsler Memory Scale – Third Edition (WMS–III), a standardized and reliable neuropsychological tool, is designed to evaluate visuospatial and memory functions [35]. The Faces Recognition, Verbal Paired Associates, Word Lists, and Spatial Span WMS–III subtests will be used to assess the immediate, delayed, and working memory [36]. For the Faces Recognition test, the participant will be required to look through all of the faces and recognize those faces later. For the Verbal Paired Associates test, the participant will be instructed to memorize several pairs of words and then will be asked to respond to the appropriate word that matches the test word. In the Word Lists test, the instructor will read out a list of words, and the participants will be instructed to repeat as many words as they can immediately and 30 minutes afterwards. During the Spatial Span test, the instructor will point to spatially located blocks in a sequential order; the participants will be instructed to touch the blocks in the same sequential order or a reversed order.

The Wechsler Adult Intelligence Scale – Third Edition (WAIS–III) is a widely used and accepted assessment tool for verbal comprehension, working memory, perceptual organization, and processing speed [37], with high reliability and validity, and is often used to differentiate individuals with cognitive deficits from those with intact cognitive functions. We will use the Digit Symbol-Coding and Matrix Reasoning tests. The Digit Symbol-Coding test consists of 9 digit-symbol pairs, and the participants will be asked to write the corresponding symbols for the given digits on the test sheet as accurately and as fast as possible. The Matrix Reasoning test requires the participants to solve missing puzzles within given matrixes, which entail the abilities of visual-spatial reasoning, abstract reasoning, visual organization, and visuospatial information processing [36].

The Useful Field of View (UFOV) is a computer-based visual test that assesses visuomotor processing speed, divided attention, and selective attention. The UFOV is the visual area over which information can be extracted from a brief glance without eye or head movements. The UFOV has good test-retest reliability and validity to assess patients with stroke [38].

The Stroop Color-Word test assesses the abilities of selective attention, inhibition, and executive function. The participants will be tested under congruent and incongruent conditions. In the congruent condition, the participant will name the color of the ink of a word, which is consistent with the written color name; whereas, in the incongruent condition, the participant will name the color of the ink, which differs from the written color name. In both conditions, the number of colors correctly named within 45 seconds will be measured, and the performance in the congruent condition will be compared with the incongruent condition.

The dual-task test evaluates the ability to shift attention between one task and another. Participants will perform the Box and Block test (BBT) while doing secondary cognitive tasks while sitting. Participants will perform the BBT using the affected and less affected hand. Two cognitive secondary tasks will be performed: (1) arithmetic task: participants will be asked to perform serial subtractions by 3 starting from 100 or random two-digit numbers (e.g., as previously described [39]); (2) tone discrimination task: participants will be presented a number of low-pitched and high-pitched tones and will calculate and remember the number of high-pitched tones during the trial. Both cognitive task performances will be recorded and the results compared against a single cognitive task performance. Participants will perform both secondary cognitive tasks while walking.

### Secondary outcomes

#### Physiological markers

The cognitive and physical related biomarkers will be collected at baseline and immediately after the intervention programs. In particular, the level of BDNF will be a biomarker after cognitive rehabilitation, whereas the antioxidative marker, blood glucose indicator, and plasma lipid levels will be evaluated as exercise-related factors. For the assessment of BDNF, blood (8 mL) will be collected in sampling tubes and centrifuged at 2000 *g* for 20 minutes. Serum will be harvested, aliquoted, and stored at – 80 °C until analysis. The automated ferric-reducing ability of plasma assay will be implemented to measure the antioxidative marker. Serum BDNF will be quantified using an enzyme-linked immunosorbent assay (Human BDNF Quantikine Immunoassay, DBD00, R&D Systems) according to the manufacturer’s instructions. This sandwich enzyme-linked immunosorbent assay is set to measure natural and recombinant human mature BDNF in serum and plasma. All assays will be performed on F-bottom 96-well plates (Nunc, Wiesbaden, Germany).

Antioxidative markers will be used to reflect the changes in oxidative stress. In particular, we will be analyzing the total antioxidant capacity. HbA_1c_ level will be tested to investigate the relationships between blood glucose level and aerobic exercise. Last, the cholesterol ratio (total cholesterol/high-density lipoprotein cholesterol) will be evaluated to reflect the lipid level in the blood.

#### ADLs and QOL

The Functional Independence Measure (FIM) and Lawton Instrumental Activities of Daily Living Scale (Lawton IADL) will be used to evaluate ADLs. The FIM assesses the dependence level of individuals with stroke to perform 18 ADLs. The score ranges from 18 to 126, and higher scores demonstrate greater independent participation in daily activities [40]. The FIM has good inter-rater reliability and validity [41].

The Lawton IADL assesses independent living skills such as shopping or managing finances [42]. The ability to perform IADLs has often been shown to decline before basic ADLs; hence, evaluating IADLs may help clinicians to identify early deterioration in physical and/or cognitive functions. The Lawton IADL scale evaluates eight activities with a score range from 0 to 8 (a higher score indicates better function).

QOL will be assessed with the Stroke Impact Scale (SIS) 3.0, EuroQoL (EQ)-5D, and Caregiver Burden (CB) scale. The SIS 3.0, which will be used to evaluate health-related QOL in the patients with stroke, uses 59 test items to assess 8 domains (strength, hand function, ADL/IADL, mobility, communication, emotion, memory and thinking, and participation/role function). Participants rate each item according to their perceived difficulty to accomplish the task during the past week. The psychometric properties of SIS in individuals with chronic stroke have been well-established [43].

The CB scale evaluates the burden of the primary caregiver of the participants. Lessening the burden of caregivers after the intervention may significantly improve the QOL for patients with stroke and their families. The CB scale measures factors related to general strain, isolation, disappointment, emotional involvement, and environment of the caregivers. The CB scale for caregivers of stroke patients has moderate-to-good test-retest reliability and construct validity [44].

QOL will be assessed by the EQ-5D questionnaire, which comprises the 5 dimensions of mobility, self-care, usual activities, pain/discomfort, and anxiety/depression. Each dimension has 3 levels: no problems, some problems, extreme problems. The score has been shown to be reliable and valid [45].

#### Physical function

Objective outcomes will include physical domains in balance, lower extremity muscle endurance and strength, mobility level, a subjective measure to assess changes in health-related physical activity, and motor impairment level. The TUG test assesses dynamic balance ability and mobility. The participants will be required to stand up from a chair, walk 3 m, turn around, walk back to the chair, and sit down. The time to complete the TUG test has been shown to be a good indicator to detect potential fallers in frail elderly individuals [46]. The test-retest reliability of TUG in individuals with stroke was excellent [47].

The 6-Minute Walk Test (6MWT) measures the maximum distance walked over 6 minutes, which assess the endurance and mobility level of the participants. The participants can rest as needed during the test. The test-retest reliability and responsiveness have been established to be high for patients with chronic stroke [48].

Accelerometers will be used to provide an objective measure of the amount of arm movements in real-life situations (i.e., the mobility level). The participants will be asked to wear an Actigraphy activity monitor (ActiGraph, Shalimar, FL, USA) on both wrists for 3 consecutive days before and after training to measure the number of moves each minute and the average counts of move per minute. The participants will be required to wear the device during the day except for doing water-based activities such as bathing or swimming. Data recorded by the Actigraphy will be analyzed with the MAHUFFE software (http://www.mrc-epid.cam.ac.uk/). The use of the Actigraphy to measure arm use and physical activity has been established in patients with stroke [49].

The International Physical Activity Questionnaires (IPAQ) is an international measure of health-related physical activity. The short-form version of the Chinese IPAQ will be used to assess changes in physical activity before and after the intervention. The reliability and validity of IPAQ have been established in 12 countries [50, 51].

The 33-item upper limb subscale of the FMA will be used to assess motor impairments. Items are scored on a 3-point ordinal scale (0 = cannot perform, 1 = performs partially, 2 = performs fully), with a total of score of 66. Proximal shoulder/elbow and distal hand/wrist subscores will be calculated. The reliability, validity, and responsiveness of the FMA, and the clinically important differences when using the FMA have been well-established in stroke patients [52, 53].

The Rivermead Mobility Index (RMI) evaluates the participant’s bed mobility, postural transfers, and walking ability. It contains a 15-item scale that includes 14 questions and 1 direct observation, with a total of score of 15. The RMI has been shown to have excellent correlation with the Functional Independence Measure (FIM) and the Barthel Index [54].

Finally, we will evaluate lower extremity muscle strength (e.g., isometric knee flexors and extensor muscle strength) and the grip strength of the affected and less affected hand using a handheld dynamometer. During the lower extremity examination, the participant will be seated upright in a chair with back support and the knee will be placed in 90° flexion. The evaluator will stabilize the participant to eliminate synergistic movements. Participants will be asked to perform a maximal isometric contraction of knee flexion and extension with the affected and less affected side. The hand dynamometer will also measure grip strength while the participant is seated with the elbow at 90° flexion. We will record the mean value of three attempts of upper and lower extremity assessments.

#### Social participation and level of depression

The participant’s level of social participation will be assessed using the Community Integration Questionnaire (CIQ). The CIQ measures 15 items relevant to home integration, social integration, and productive activities [55]. The total score ranges from 0 to 29 points. The CIQ has been tested on various populations with acquired brain injuries, and test-retest reliability was excellent for chronic stroke patients with aphasia [55, 56]. The Geriatric Depression Scale (GDS) is a self-administered questionnaire used to evaluate mood and depressive symptoms, with each question requiring a yes or no response. A short form of the GDS will be used in this study to assess the participants. The reliability, internal consistency, and validity have been established to be good to excellent in patients with stroke [57].

### Analyses

We will use the chi square (χ^2^) test and analysis of variance to analyze differences in baseline characteristics and baseline outcome measures among the groups. Mixed-factor repeated-measures analysis of variance will be used to determine the intervention effect in the three groups. The within-subject factor will be time (pretest, posttest, and follow up), and the between-subjects factor will be group (COG, AE, and SEQ). Tukey’s test will be performed if a group × time interaction or a main effect is observed. Statistical significance will be set at 0.05 for all comparisons. In addition to *p* values, *η* ^2^ will also be calculated to determine the group difference for each outcome measure. Data analysis will be performed using PASW Statistics 18.0 software (SPSS Inc., Chicago, IL, USA).

## Data management and quality

A paper-based assessment form will be used by independent outcome evaluators to record the outcome measures. A trained research assistant who is independent of the trial will manually enter the data into an electronic database; another independent research assistant will check data quality and accuracy (e.g., range checks, checking for missing data for required data points). The data collection sheets and signed informed consent forms will be stored in a locked cabinet, and the electronic database will be password-protected. Blood samples will be collected by nurse practitioners from a peripheral vein in the patient’s forearm. A standard operating procedure (SOP) for blood sample collection, handling, and storage will be established to ensure data stability.

## Adverse event monitoring and reporting

Adverse events will be carefully monitored during the trial. Expected serious events include recurrent stroke or transient ischemic attack (TIA), myocardial infarction, fracture, pulmonary embolism, and death. Expected non-serious events include falls, severe pain or muscle soreness, excessive blood pressure responses, and dizziness. The interventionists will closely monitor the participants, especially during the aerobic exercise training to minimize the risk of any adverse event. Adverse events will be reported immediately to the responsible physician and the principle investigator, and will then be reported to the IRB in accordance with the procedures of Chang Gung University, Taoyuan, Taiwan.

## Discussion

Advances in stroke management, including intravenous thrombolysis, endovascular therapy with thrombectomy, organized care for ischemic stroke, and extensive control of risk factors for stroke prevention, have resulted in a gradual decrease in the mortality rate and an increase in life expectancy after stroke. The management of neurologic deficits and declined function after stroke has become a critical issue. In contrast to the successful pharmaceutical management of risk factors and great emphasis on motor function recovery, interventions for cognitive impairment remain under development.

Cognitive deficits after stroke are strongly associated with functional disability, institutionalization rate, and the risk of developing dementia. Treatment to reserve cognitive ability is emerging, and attention in recent decades has shifted toward this less visible cognitive impairment. There is no consensus about pharmacologic interventions [58], and the use of medicines alone for cognitive decline after stroke is not highly recommended. Accordingly, structuring non-pharmacologic interventions to increase or reserve patients’ cognitive capacity is important.

Aerobic exercise is considered to be effective in improving cognitive function and has the advantages of being simple, convenient, efficient, inexpensive, and without severe adverse effects [59]. Targeted cognitive training after stroke has also been shown to promote cognition [9]. The recent use of combined therapy has increasingly attracted attention. Aerobic exercise combined with cognitive training, which has not been studied in patients with stroke, might offer a new breakthrough in training cognitive function after stroke. A systematic review has proposed that despite the increase in the number of studies that have focused on training for cognitive function after stroke, the lack of high-quality research has prevented the development of evidence-based recommendations for clinical practice [11]. The review also recommends that the optimal intervention approach should use a large sample, an adequate dose of treatment, and explore changes in physiological markers, ADLs, and QOL after training. The long-term retention of the training benefits should also be assessed. This trial represents endeavor toward this possibility.

The objectives of this proposed trial are to determine the treatment effects of a sequential combination of aerobic exercise and cognitive training, compared with exercise or cognitive training alone, on cognitive function, physiological markers, daily function, QOL, physical function, and social participation. A notable difference between our study and previous studies is that this trial will investigate the physiological biomarkers and a variety of outcomes related to patient’s ADL, QOL, and social participation. The serum BDNF level has been selected to evaluate whether cognitive training can upregulate the neurotrophic and vascular growth factors, and the relationship between cognitive performance and serum BDNF levels will be assessed.

Meanwhile, because the fundamental goal of rehabilitation is to improve everyday functioning, determining the effect of cognitive training on ADL, QOL, and social participation is important. The establishment of a core set of outcome measures would be particularly helpful because these outcomes reflect the “real-life” significance of cognitive training. The trial also aims to determine the long-term treatment effects of different training protocols on these outcome measures. A 12-week intervention and a 24-week follow-up assessment can potentially provide reliable evidence on the long-term effects of the therapeutic regimen of cognitive training in stroke survivors.

Limitations of the design and implementation of the trial must also be considered. In this trial, we designed the cognitive training and aerobic exercise training groups as comparisons to a sequential combination of aerobic exercise and cognitive training rather than including a passive control group. Our primary aim in this trial is a parallel comparison of three experimental interventions rather than a comparison with conventional therapy. Hence, no control group that receives traditional cognitive training will be included.

With the global population aging and the increasing number of stroke survivors, there is growing urgency to identify the most effective methods to promote cognitive function. We believe that the information garnered from this trial will provide evidence for the potential synergistic intervention on cognitive function in stroke survivors, which is crucial to health care professionals involved in cognitive rehabilitation.

## Trial status

Currently recruiting.

## Additional file

Additional file 1: SPIRIT 2013 Checklist: recommended items to address in a clinical trial protocol and related documents. (DOC 120 kb)

## Abbreviations

6MWT: 6-Minute Walk Test
ADLs: Activities of daily living
AE: Aerobic exercise
BBT: Box and Block Test
BDNF: Brain-derived neurotrophic factor
CB: Caregiver Burden
CDR: Clinical Dementia Rating Scale
CIQ: Community Integration Questionnaire
COG: Cognitive training
ELISA: Enzyme-linked immunosorbent assay
FIM: Functional Independence Measure
FMA: Fugl-Meyer Assessment
GDS: Geriatric Depression Scale
IPAQ: International Physical Activity Questionnaires
IRB: Institutional Review Board
Lawton IADL: Lawton Instrumental Activities of Daily Living Scale
MMSE: Mini-Mental State Examination
MoCA: Montreal Cognitive Assessment
MRS: Modified Rankin Scale
NIHSS: National Institutes of Health Stroke Scale
QOL: Quality of life
RCT: Randomized controlled trial
RMI: Rivermead Mobility Index
SEQ: Sequential combination of aerobic exercise and cognitive training
SIS: Stroke Impact Scale
TUG: Timed Up and Go
UFOV: Useful Field Of View
WAIS: Wechsler Adult Intelligence Scale
WMS: Wechsler Memory Scale
